# Detection of* Yersinia enterocolitica* in Retail Chicken Meat, Mashhad, Iran

**DOI:** 10.1155/2018/1286216

**Published:** 2018-04-19

**Authors:** Khadigeh Sirghani, Tayebeh Zeinali, Abdollah Jamshidi

**Affiliations:** ^1^Food Hygiene and Quality Control, Faculty of Veterinary Medicine, Ferdowsi University of Mashhad, Mashhad, Iran; ^2^Social Determinants of Health Research Center, Faculty of Health, Birjand University of Medical Sciences, Birjand, Iran; ^3^Department of Food Hygiene, Faculty of Veterinary Medicine, Ferdowsi University of Mashhad, Mashhad, Iran

## Abstract

Poultry meat is one of the most important sources of infection of* Yersinia* spp. for humans. The aim of the present study was to evaluate the incidence of* Yersinia enterocolitica* in chicken meat by using culture method on selective medium and confirmation by PCR assay. Also, biochemical methods were used for biotyping. A total of 100 chicken thigh meat samples were collected randomly from retail outlets in Mashhad, Iran. Samples were enriched in Peptone-Sorbitol-Bile (PSB) broth and then cultured on Cefsulodin-Irgasan-Novobiocin (CIN) agar containing antibiotics supplement. The DNA was extracted from suspected colonies of* Yersinia* spp. and then PCR test using specific primers for 16S rRNA gene of* Yersinia enterocolitica* was performed. In this study, 30% of chicken meat was contaminated with* Yersinia *spp. by culture method and 25% of chicken meat was contaminated with* Yersinia enterocolitica*. Biotyping of isolated colonies showed that all of the isolates belonged to biotype 1A. Culture and detection of* Yersinia* spp. from food samples traditionally take 4 days. Due to high accuracy and speed of PCR assay, it is a good alternative method for microbiological techniques. In conclusion, poultry meat can act as a source of* Y. enterocolitica* and could be considered as a public health hazard.

## 1. Introduction

*Yersinia enterocolitica* as a member of Enterobacteriaceae family is a Gram-negative non-spore-forming rod. It is a psychrotrophic bacterium and able to survive and multiply at refrigerator temperature [1].* Y. enterocolitica *is an enteric pathogen which commonly causes acute enteritis associated with fever, bloody diarrhea, and inflammation of lymph nodes which frequently leads to unnecessary laparotomy due to pseudoappendicitis in humans [2].

In developing countries like Iraq [3], Iran [4], and Nigeria [5], the prevalence of gastrointestinal illness is highlighted including yersiniosis which highlights the major underlying food safety problems in low- and middle-income countries.

Young children and infants are the most susceptible groups which are at risk of infection [6]. In Iran, little information is available about annual infections of* Y. enterocolitica*. In Southeast Asian countries, few reports are available on the incidence of yersiniosis [7, 8]. Contaminated food is one of the main sources of yersiniosis in humans [9].

*Y. enterocolitica* is widely distributed in the nature and animals; food and environment are routinely contaminated with this organism [10]. Major reservoir of* Y. enterocolitica* is swine [11]. Furthermore,* Y. enterocolitica* has been frequently isolated from poultry [12] and ready-to-eat foods [13]. However, all strains of* Y. Enterocolitica* are not pathogenic to humans but some strains such as biotypes 1B/O:8, 2/O:5,27, 2/O:9, 3/O:3, and 4/O:3 are human pathogens [13].

There are a low number of studies on* Y. Enterocolitica* in Iran and there is no program to check the bacterium routinely. Because of the limited number of studies on* Y. enterocolitica* especially in northeast of Iran, the actual incidence of organism remains unknown. Therefore, the aims of this study were (i) determining the contamination rate of raw chicken meat with* Y. enterocolitica* and (ii) identifying the common biotypes of* Y. enterocolitica* which are currently present in retail chicken meat.

## 2. Materials and Methods

### 2.1. Sample Collection

A total of 100 raw chicken thigh samples as a representative of chicken meat were obtained by stratified random sampling method from different supermarkets and retail outlets in northeast of Iran, from January 2017 until July 2017. The samples were collected in sterile bags and immediately transported to the laboratory at refrigeration temperature (3°C).

### 2.2. Isolation and Identification of* Y. enterocolitica*

A 10 g aliquot of each sample was cut using sterile scissors and tissue forceps and put into sterile Stomacher bags containing 90 mL of Peptone-Sorbitol-Bile (PSB) broth (Sigma-Aldrich, Germany) and homogenised by bag mixer for 2 minutes. The samples diluted in PSB were incubated at 25°C in a shaker incubator. Thereafter, 0.5 ml of the enriched samples was mixed with 4.5 ml of potassium hydroxide (KOH) 0.25% and streaked onto CIN agar (Merck, Darmstadt, Germany) plates supplemented with Cefsulodin-Irgasan-Novobiocin antibiotics (Merck, Darmstadt, Germany) [14]. After 24–48 h of incubation at 30°C, small (1-2 mm diameter) colonies with deep red center and sharp border surrounded by clear colorless zone with entire edge in CIN agar plates were selected. Colonies with negative Gram staining were selected for biochemical tests including catalase, oxidase, and urease.* Yersinia *spp. are oxidase-negative and catalase- and urease-positive.

### 2.3. DNA Extraction and PCR Assay

The DNA was extracted from purified suspected colonies using conventional boiling method [15]. Amplification of 16s rRNA was performed in final volume of 20 *μ*l, containing 1 *μ*l (10 picomol) of forward (5′-AATACCGCATAACGTCTTCG-3′) and reverse (5′-CTTCTTCTGCGAGTAACGTC-3′) primer (Macrogen, Republic of Korea), 2 *μ*l DNA template, 10 *μ*l of master mix (Ampliqon, Denmark), and 6 *μ*l nuclease-free deionized distilled water. Thermal cycler program was as follows: Initial denaturation was at 94°C for 5 min and final extension at 72°C for 7 min. Denaturation was at 94°C for 45 sec, annealing at 62°C for 45 sec, and extension at 72°C for 45 sec in 36 cycles.

*Yersinia enterocolitica* (ATCC 9610) was used as positive control and for negative control, nuclease-free deionized water was used. PCR products were separated on a 1.5% agarose gel which was prestained by green viewer and photo-documented under UV illumination.

### 2.4. Biotyping

To determine the biotype of isolates, esculin, indole, and lipase activity were investigated. Also, fermentation test of salicin, trehalose, sorbose, ornithine decarboxylase, inositol, and xylose was performed [16].

## 3. Results

**Figure 1:**
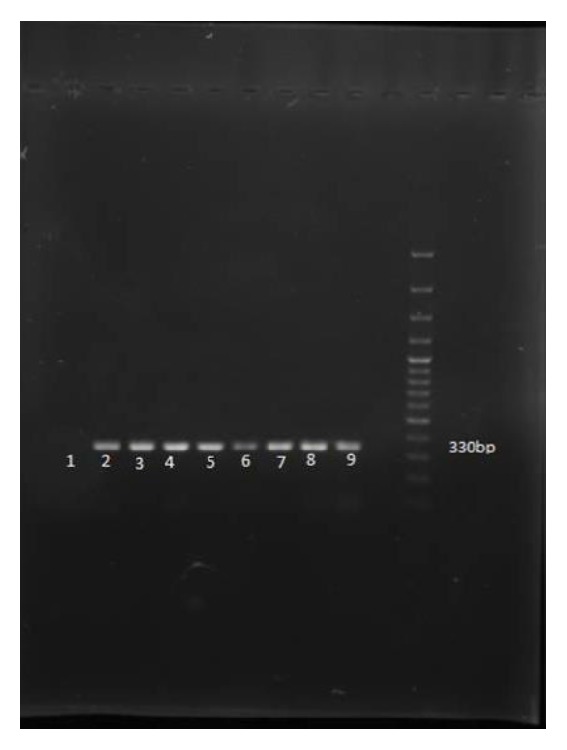
PCR amplification of 16s rRNA gene (330 base-pair) of* Y. enterocolitica. *Lane 1: negative control; Lanes 2 to 8: positive samples; Lane 9: positive control; Lane 12: 100 base pairs plus DNA Ladder.

According to conventional culture method, 30 out of 100 (30%) raw chicken meat samples were contaminated with* Yersinia* spp. Amplification of 16s rRNA gene identified 25 isolates as* Yersinia enterocolitica*. In other words, 25% of chicken meat samples were contaminated with* Yersinia enterocolitica* in northeast of Iran.  Figure 1 shows the amplification of 16s rRNA gene in samples. In biotyping test, all of the 25 positive isolates belonged to biotype 1A.

## 4. Discussion

One of the main problems of detecting* Y. Enterocolitica* in food is the presence of high number of background bacteria. Use of enrichment step helps in detecting this bacterium. Different enrichment procedures were used in other studies such as cold enrichment and use of ITC medium. Damme et al. (2013) with using of PSB at 25°C obtained more positive results than cold enrichment. In the present study, samples were enriched in PSB at 25°C. One of the helpful items to suppress background microorganisms is the use of alkaline treatment.* Y. enterocolitica* can tolerate weak alkaline treatment, but background flora such as* Pseudomonas* and* Proteus* will be suppressed [17]. In other studies in Iran, India, Egypt, and China, 0% to 30% of chicken meat was contaminated with* Y. enterocolitica *[16, 18–21].

A study in Argentina reported a higher prevalence of *Y*. enterocolitica-positive chicken eggshell samples (38.65%) if compared to our results [22]. In France, 5.2% of poultry were contaminated with* Y. Enterocolitica* and all of the isolates were detected as biotype 1A [23].

In a study conducted in Spain, 65% of chicken carcasses were contaminated with Yersinia spp., and 52 out of 68 isolates were identified as* Y. enterocolitica. *Biotyping of* Y. enterocolitica* revealed that 86.5% of isolates belonged to biotype 1A and three (5.8%) to biotype 3 [24].

Momtaz et al. (2013) detected a lower prevalence of *Y*. enterocolitica-positive raw chicken meat samples (18.33%) if compared to our study. The isolates belonged to biotypes 2, 3, 4, and 5 and no 1A isolates were detected. This result was not in agreement with our results [25].

Determination of pathogenicity of* Y. enterocolitica* strains is based on the presence of some specific virulence genes including* ail, virF, yadA, inv, myfA, ystA, ystB, tccC, hreP, fepA, fepD, fes, ymoA,* and* sat* [16].

Our findings agreed with those of other researchers that biotype 1A is the most prevalent, or even the only isolated biotype, in poultry and meat [16, 23, 26–28]. It must be noted that some studies report that strains of biotype 1A can occasionally act as opportunistic pathogens and cause extraintestinal infections [29]. Indeed, biotype 1A caused two gastrointestinal outbreaks. To determine the pathogenicity of 1A strains, researchers compared genome sequence of two nosocomial and environmental strains with two virulent strains including biotypes 1B and 4. Interestingly, biotype 1A had common genes with pathogenic biotypes 1B and 4. Moreover, 1A strains, despite the lack of some classical virulence marker such as* ail* adhesion, the* ystA* enterotoxin, and the virulence-associated protein C, still carry some genes encoding such known and suspect virulence-associated proteins like the* ystB* enterotoxin, the* InvA* invasin, the mucoid* Yersinia* factor MyfA, and the enterochelin utilisation* fepBDGC*/*fepA*/*fes* gene cluster [30]. In a study, 128 clinical strains of* Y. enterocolitica* were characterized in Switzerland. 58.6% of these strains were identified as biotype 2, 3, or 4 and harbor* ail* gene. One of the 1A strains was also* ail*-positive [31].

It must be considered that cross-contamination of cooked and raw meat can occur from the interior surfaces of household refrigerators [32] or through storage containers. Some risk factors for human's yersiniosis are inappropriate food handling, processing, and storing such as undercooked meats or cross-contamination of contaminated meats or surfaces to other food [17].

## 5. Conclusion

Our study indicates that consumption of chicken meat presents a low risk of pathogenic* Y. enterocolitica* for humans. Although all of the strains were biotype 1A, the incidence of* Y. enterocolitica* is relatively high and this risk should not be ignored.

## Data Availability

The data used to support the findings of this study are available from the corresponding author upon request.

## References

[1] Annamalai T., Venkitanarayanan K. (2005). Expression of major cold shock proteins and genes by Yersinia enterocolitica in synthetic medium and foods. *Journal of Food Protection*.

[2] Vlachaki E., Tselios K., Tsapas A., Klonizakis J. (2007). Yersinia enterocolitica O:3 mesentic lymphadenopathy in an apparently healthy adult. *The Netherlands Journal of Medicine*.

[3] Kanan T. A., Abdulla Z. A. (2009). Isolation of Yersinia spp. from cases of diarrhoea in Iraqi infants and children. *Eastern Mediterranean Health Journal*.

[4] Soltan-Dallal M. M., Moezardalan K. (2004). Frequency of Yersinia species infection in paediatric acute diarrhoea in Tehran. *Eastern Mediterranean Health Journal*.

[5] Okwori A. E. J., Martínez P. O., Fredriksson-Ahomaa M., Agina S. E., Korkeala H. (2009). Pathogenic Yersinia enterocolitica 2/O:9 and Yersinia pseudotuberculosis 1/O:1 strains isolated from human and non-human sources in the Plateau State of Nigeria. *Food Microbiology*.

[6] Rosner B. M., Stark K., Werber D. (2010). Epidemiology of reported Yersinia enterocolitica infections in Germany, 2001-2008. *BMC Public Health*.

[7] Ananchaipattana C., Hosotani Y., Kawasaki S. (2012). Bacterial contamination of soybean curd (Tofu) sold in Thailand. *Food Science and Technology Research*.

[8] Ananchaipattana C., Hosotani Y., Kawasaki S. (2012). Prevalence of foodborne pathogens in retailed foods in Thailand. *Foodborne Pathogens and Disease*.

[9] Bonardi S., Bruini I., D'Incau M. (2016). Detection, seroprevalence and antimicrobial resistance of Yersinia enterocolitica and Yersinia pseudotuberculosis in pig tonsils in Northern Italy. *International Journal of Food Microbiology*.

[10] Fredriksson-Ahomaa M., Korkeala H. (2003). Low occurrence of pathogenic *Yersinia enterocolitica* in clinical, food, and environmental samples: a methodological problem. *Clinical Microbiology Reviews*.

[11] Fondrevez M., Labbé A., Houard E., Fravalo P., Madec F., Denis M. (2010). A simplified method for detecting pathogenic Yersinia enterocolitica in slaughtered pig tonsils. *Journal of Microbiological Methods*.

[12] Dallal M. M. S., Doyle M. P., Rezadehbashi M. (2010). Prevalence and antimicrobial resistance profiles of *Salmonella* serotypes, *Campylobacter* and *Yersinia* spp. isolated from retail chicken and beef, Tehran, Iran. *Food Control*.

[13] Rahman A., Bonny T. S., Stonsaovapak S., Ananchaipattana C. (2011). *Yersinia enterocolitica*: epidemiological studies and outbreaks. *Journal of Pathogens*.

[14] Damme I. V., Berkvens D., Botteldoorn N. (2013). Evaluation of the ISO 10273:2003 method for the isolation of human pathogenic Yersinia enterocolitica from pig carcasses and minced meat. *Food Microbiology*.

[15] Zeinali T., Jamshidi A., Rad M., Bassami M. (2015). A comparison analysis of Listeria monocytogenes isolates recovered from chicken carcasses and human by using RAPD PCR. *International Journal of Clinical and Experimental Medicine*.

[16] Ye Q., Wu Q., Hu H., Zhang J., Huang H. (2016). Prevalence and characterization of Yersinia enterocolitica isolated from retail foods in China. *Food Control*.

[17] Tan L. K., Ooi P. T., Thong K. L. (2014). Prevalence of Yersinia enterocolitica from food and pigs in selected states of Malaysia. *Food Control*.

[18] Aghamohammad S., Gholami M., Dabiri H. (2015). Distribution and Antimicrobial Resistance Profile of Yersinia Species Isolated From Chicken and Beef Meat. *International Journal of Enteric Pathogens*.

[19] Shanmugapriya S., Senthilmurugan T., Thayumanavan T. (2014). Genetic diversity among Yersinia enterocolitica isolated from chicken and fish in and around Coimbatore city, India. *Iranian Journal of Public Health*.

[20] Shabana S., Khalil S., Hegazy A. (2015). Molecular Characterization of Yersinia Enterocolitica Isolated From Chicken Meat Samples. *Alexandria Journal of Veterinary Sciences*.

[21] Anju P. Detection of Salmonella and Yersinia spp. in uncooked retail chicken meat in Kerala by multiplex PCR.

[22] Favier G. I., Escudero M. E., De Guzmán A. M. S. (2005). Genotypic and phenotypic characteristics of Yersinia enterocolitica isolated from the surface of chicken eggshells obtained in Argentina. *Journal of Food Protection*.

[23] Esnault E. (2013). Yersinia enterocolitica prevalence, on fresh pork, poultry and beef meat at retail level. *in France*.

[24] Capita R., Alonso-Calleja C., Prieto M., García-Fernández M. D. C., Moreno B. (2002). Incidence and pathogenicity of Yersinia spp. isolates from poultry in Spain. *Food Microbiology*.

[25] Momtaz H., Rahimian M. D., Safarpoor Dehkordi F. (2013). Identification and characterization of Yersinia enterocolitica isolated from raw chicken meat based on molecular and biological techniques. *Journal of Applied Poultry Research*.

[26] Floccari M. E., Carranza M. M., Parada J. L. (2000). *Yersinia enterocolitica* biogroup 1A, serotype O:5 in chicken carcasses. *Journal of Food Protection*.

[27] Lim S. Y., Yoon S. K. (2000). Characteristics of Yersinia enterocolitica isolated from frozen foods. *Korean Journal of Food Science and Technology*.

[28] Ramírez E. I. Q., Vázquez-Salinas C., Rodas-Suárez O. R., Pedroche F. F. (2000). Isolation of Yersinia from raw meat (pork and chicken) and precooked meat (porcine tongues and sausages) collected from commercial establishments in Mexico City. *Journal of Food Protection*.

[29] de Boer E., Seldam W. M., Oosterom J. (1986). Characterization of Yersinia enterocolitica and related species isolated from foods and porcine tonsils in the Netherlands. *International Journal of Food Microbiology*.

[30] Batzilla J., Heesemann J., Rakin A. (2011). The pathogenic potential of *Yersinia enterocolitica* 1A. *International Journal of Medical Microbiology*.

[31] Fredriksson-Ahomaa M., Cernela N., Hächler H., Stephan R. (2012). Yersinia enterocolitica strains associated with human infections in Switzerland 2001-2010. *European Journal of Clinical Microbiology & Infectious Diseases*.

[32] Jackson V., Blair I. S., McDowell D. A., Kennedy J., Bolton D. J. (2007). The incidence of significant foodborne pathogens in domestic refrigerators. *Food Control*.

